# Circular RNAs: The Novel Actors in Pathophysiology of Spinal Cord Injury

**DOI:** 10.3389/fnint.2021.758340

**Published:** 2021-10-14

**Authors:** Cynthia Sámano, Miranda Mladinic, Graciela L. Mazzone

**Affiliations:** ^1^Departamento de Ciencias Naturales, Universidad Autónoma Metropolitana, Unidad Cuajimalpa, Cuajimalpa de Morelos, Mexico; ^2^Department of Biotechnology, University of Rijeka, Rijeka, Croatia; ^3^Instituto de Investigaciones en Medicina Traslacional (IIMT), CONICET-Universidad Austral, Buenos Aires, Argentina

**Keywords:** spinal cord injury, circRNAs, biomarkers, non-coding RNA, circRNAs in central nervous system

## Abstract

Spinal Cord Injury (SCI) can elicit a progressive loss of nerve cells promoting disability, morbidity, and even mortality. Despite different triggering mechanisms, a cascade of molecular events involving complex gene alterations and activation of the neuroimmune system influence either cell damage or repair. Effective therapies to avoid secondary mechanisms underlying SCI are still lacking. The recent progression in circular RNAs (circRNAs) research has drawn increasing attention and opened a new insight on SCI pathology. circRNAs differ from traditional linear RNAs and have emerged as the active elements to regulate gene expression as well as to facilitate the immune response involved in pathophysiology-related conditions. In this review, we focus on the impact and possible close relationship of circRNAs with pathophysiological mechanisms following SCI, where circRNAs could be the key transcriptional regulatory molecules to define neuronal death or survival. Advances in circRNAs research provide new insight on potential biomarkers and effective therapeutic targets for SCI patients.

## Introduction

Circular RNAs (circRNAs) are endogenous single-stranded RNA molecules produced by circularization events (Guo et al., 2014; Wang et al., 2014), shown to be very stable and conserved functional molecules, highly expressed in the central nervous system (CNS; Memczak et al., 2013; Rybak-Wolf et al., 2015). circRNAs play crucial regulatory roles in gene expression at the posttranscriptional level (Zhang et al., 2013; Chen, 2020) and are closely associated with multiple neurodegenerative diseases, especially at the level of the neuro-immune pathways (Qu et al., 2020; Xu et al., 2021). Therefore, it is possible that circRNAs may also play important role in molecular events involved in spinal cord injury (SCI) pathology.

SCI is a common and serious neurological condition with social and economic consequences (Sweis and Biller, 2017). Either traumatic or non-traumatic, SCIs start with primary insult, followed by subsequent secondary pathological events that amplify spinal neurodegeneration (Quadri et al., 2020). Current treatments exhibit limited efficacy for complete functional recovery after SCI (Martirosyan, 2021), mostly because of the poor understanding of molecular events underlying secondary injury.

circRNAs have been recently shown to be involved in the pathogenesis and promotion of neuro-inflammation, including the altered expression of circRNAs in critical stages of SCI physiopathology (Qin et al., 2019; Zhou et al., 2019). The aim of this review is to highlight the intriguing expression of circRNAs after SCI where they could play a paramount role in neurodegeneration and offer new therapeutic targets.

## Characteristics of circRNAs

Despite knowing about circRNAs for at least 20 years, recent bioinformatic tools have allowed a rediscovering their endogenous role (Patop et al., 2019) in a wide spectrum of biological (Rybak-Wolf et al., 2015; Mahmoudi and Cairns, 2019) and pathological conditions in CNS (Kumar et al., 2017; Qu et al., 2020). circRNAs are well conserved and exhibit cell-type or tissue specificity across the animal kingdom (Jeck and Sharpless, 2014; Rybak-Wolf et al., 2015). Most circRNAs are generated by back-splicing events with a wide range of sizes (from 100 nt to over 10,000 nt), arising from exonic, intronic, intergenic untranslated regions (UTRs) or tRNAs (Jeck et al., 2013; Noto et al., 2017). They represent covalently closed circles with neither 5′ to 3′ polarity nor a polyadenylated tail, giving more resistance to degradation by exonucleases with half-lives exceeding 48 h (Chen and Yang, 2015; Xiao et al., 2020). Most circRNAs do not undergo translation (Guo et al., 2014), though some studies have provided evidence for a subset of circRNAs which can be efficiently translated (Legnini et al., 2017; Pamudurti et al., 2017; Bagchi, 2018). circRNAs have been involved in modulation of alternative RNA splicing or transcription (Salzman, 2016) by competing for endogenous RNAs (ceRNAs; Li et al., 2017), sequestering miRNAs (Hansen et al., 2013), acting as protein scaffolds (Salvatori et al., 2020) and regulating gene expression by interacting with RNA binding proteins (Kristensen et al., 2019).

## circRNAs in CNS

The circRNAs that are widely expressed in the mammalian brain (Rybak-Wolf et al., 2015; Hanan et al., 2017) have been suggested to participate in neurodevelopment (Hansen et al., 2013; Memczak et al., 2013), neuronal proliferation, and differentiation (Chen and Schuman, 2016; Yang et al., 2019; Suenkel et al., 2020). For example, a significant increase of certain circRNAs has been associated with myelination and neural maturation in the brain and spinal cord during postnatal development (Di Agostino et al., 2020), revealing the key role of circRNAs also in motoneuron development in cortex, brainstem, or spinal cord. These results indicate circRNAs as possible valuable therapeutic targets for different degenerative motoneuron diseases (Vangoor et al., 2021). Interestingly, exosomal circRNAs (exo-circRNAs) have been also indicated as novel gene expression regulators and cell-to-cell communicators, with a possible role in physiopathological processes in CNS (Zhao et al., 2018; Wang et al., 2019; Zhang et al., 2019). Indeed, cell type differentiation and development are highly regulated processes mediated by circRNAs (Gapp et al., 2014; Meng et al., 2019). Recent investigations have shown that circRNAs regulate neuronal and glial lineages (astrocytes, oligodendrocytes, and microglia) by functioning as gene transcription regulators (Curry-Hyde et al., 2020; Salvatori et al., 2020; Suster and Feng, 2021). Another role of the circRNAs has been reported during development of the neocortex where they regulate spontaneous neuronal differentiation and are important to maintain the pool of cell progenitors (Suenkel et al., 2020). The spatiotemporal expression and function of lineage-specific circRNAs partly underlie the regulation of transcription factors to induce neuronal and glial differentiation and proliferation (Yang et al., 2019). Moreover, since circRNAs exhibit differential expression in different brain cells and areas, they could also represent a valuable tool for the development of novel targeted markers for gliomas progression and tumorigenicity (Sun et al., 2020).

On the other hand, several studies have demonstrated that circRNAs are closely related to human diseases. For example, the best characterized and most expressed circRNA in the mammalian brain, known as CDR1as/ciRS-7, was reported as the first miRNA sponge and negative regulator of the miR-7, showing developmental and tissue stage-specific expression (Memczak et al., 2013). Early reports showed that CDR1as/ciRS-7 is highly expressed in brain tissue, neuroblastoma, astrocytoma (Hansen et al., 2013; Panda, 2018), and that strongly regulated the progression of Parkinson and Alzheimer disease (Lukiw, 2013). Recently, in post-mortem brain analysis it was demonstrated that the levels of circRNAs were increased at the substantia nigra region of healthy but not of Parkinson disease patients, showing the age-dependent accumulation, while the circRNAs levels were increased related to oxidative stress, with a unique tissue-specific profile (Hanan et al., 2020). Indeed, by performing deep RNA-seq of brain tissues from substantia nigra, amygdala, and medial temporal gyrus of Parkinson disease patients and controls, the long noncoding RNAs (lncRNAs) candidates with high relevance to disease pathology highly correlated to p53 expression (Simchovitz et al., 2020), a core transcription factor that can regulate the expression of several noncoding RNAs (Marín-Béjar et al., 2013). These interesting results highlight the possible regulatory functions of lncRNA in aging and neurodegeneration disease. Moreover, a significant increase of circRNA expression has been associated with myelination and neural maturation processes in the brain and spinal cord, during a critical period of postnatal development (Di Agostino et al., 2020). In summary, circRNAs are indicated as important regulators in the brain and spinal cord development, aging, and neurodegenerative diseases and are suggested as potential biomarkers and drug targets (Chen B. J. et al., 2019).

## circRNAs in SCI

By GO (Gen Ontology) and KEGG (Kyoto Encyclopedia of Genes and Genomes) analysis, dysregulation of circRNAs expression profiles have been found during the immediate, intermediate, and acute phases of secondary traumatic SCI (Qin et al., 2019; Zhou et al., 2019; Liu et al., 2020; Chen et al., 2021; Xu et al., 2021). Indeed, the potential function of circRNAs after acute traumatic SCI in rats were predicted by bioinformatic analysis on the three criteria (cellular components, biological process, and molecular functions), revealing the upregulated circRNAs were associated with nuclear proteins while downregulated circRNAs to proteins active in the cytoplasm (Zhou et al., 2019) and proposing that circRNAs may participate through different molecular mechanisms in SCI pathology. Moreover, the potential circRNA-miRNA-mRNA crosstalk has been proposed to regulate pathologic mechanisms after traumatic SCI. Thus, the circRNA.7079 has been related to apoptosis in an early phase of SCI in mice, *via* mmu-miR-6953-p sponge (Zhou et al., 2019). Peng et al. (2020) tried to elucidate the role of circRNAs in SCI, through the construction of circRNA-miRNA-mRNA network by microarray data and Gene Expression Ominibus (GEO), identifying the circRNA_014620 to be significantly upregulated after traumatic SCI, together with the expression of miR-223-3p and miR-182 in the final circRNA-miRNA-hub-gene-axis. Among the hub genes are DDIT4 (DNA damage-inducible transcript 4), related to neuroprotective or toxic effects under ischemic injury and oxidative stress (Li et al., 2017), EZR (ezrin), and STAT3 (signal transducing activator of transcription-3; Renault-Mihara et al., 2017), suggesting their participation in the inflammatory response and nerve regeneration (Peng et al., 2020). Another study showed that the cicRNA.7079 knockdown enhanced apoptosis in NSC-34 motor neurons (Yao et al., 2020).

Through KEGG analysis the enriched circRNAs following SCI were related to Peroxisome Proliferator-Activated Receptors (PPAR) and Extracellular Matrix-receptor (ECM) interaction pathways and glycosphingolipid biosynthesis (Zhou et al., 2019; Yao et al., 2020). Furthermore, the enriched circRNAs-miRNAs networks have been involved in the modulation of signaling pathways after SCI, such as the AMP-activated protein kinase (AMPK; Qin et al., 2019), as well as calcium-related, JAK-STAT, and MAPK signaling pathways (Liu et al., 2020). Many of these pathways play a pivotal role in the regulation of cell energy homeostasis, signal neuronal transduction, neuron apoptosis, differentiation, proliferation, long-term potentiation, immunity, and inflammation (Wang et al., 2018; Qin et al., 2019; Zhou et al., 2019; Liu et al., 2020). It is also very promising that knockdown of circRNA_01477 significantly inhibited astrocyte proliferation and migration after experimental SCI in rats (Wu et al., 2019) and that disrupting circRNA-2960 expression promoted recovery of tissues affected by secondary SCI damage (Chen et al., 2021). Nonetheless, the potential mechanism by which circRNAs impact SCI is not fully understood. Recent studies reveal the interesting role of non-coding RNA that influence several aspects of cell development at the cortex, brainstem, or spinal cord and may comprise valuable therapeutic targets for different degenerative disease (Hanan et al., 2020; Simchovitz et al., 2020; Vangoor et al., 2021). Although altered circRNAs have been closely linked with miRNA-mRNA networks in biological processes and signaling pathways during SCI progression in rodents, future research is necessary to clarify the cell-specific inflections, especially related to neurodegeneration and neuroinflammation in humans.

Interestingly, several reports have shown differential expression profiles of circRNAs after SCI, showing the complexity of the issue. Namely, it is important to consider that the dynamic time-course changes in circRNA profiles may be influenced by diverse factors: (i) the type of SCI model (rat or mice); (ii) the site of lesion; (iii) the method of detection (microarray, RNA-seq and/or bioinformatic analysis); and (iv) the specific time points post-trauma when the circRNAs are monitored. These factors could modulate the circRNAs levels, indicating the different regulatory roles of circRNAs in the physiopathology of SCI (Weng et al., 2019; Wu et al., 2019; Zhou et al., 2019; Li et al., 2020; Liu et al., 2020; Peng et al., 2020; Yao et al., 2020; Zhao et al., 2020; Peng et al., 2021). In this sense, to our knowledge, Wu et al. (2019) published the first study about the expression changes of circRNAs after SCI in rats (days 0, 1, 3, 7, 14, 21, and 28). The systematic evaluation by RNA-seq analysis, showed the 360 circRNAs being differentially expressed, 94% of which decreased from day 3 onward. In fact, according to the reports published on humans and rats, the switching event that mostly influences the circRNAs changes usually happens at 3–5 days post-injury, when neutrophil infiltration and microglia activation prevail (Fleming et al., 2006; Kjell and Olson, 2016; Bradbury and Burnside, 2019; Wu et al., 2019). Moreover, Wu et al. (2019) reported that the circRNA_01477/miR-423-5p network may be a key regulator in the regeneration mechanism following SCI (Wu et al., 2019).

The altered expression of circRNAs has been also reported to occur in the cerebral cortex after traumatic brain injury (TBI), where the upregulation of circRNAs (chr8_87, 859, 283–87, 904, 548) promoted neuro-inflammation by increasing the CXCR2 protein by sponging miRNA mmu-let-7a-5p (Chen Z. et al., 2019). Also in the cerebrovascular Moyamoya disease (MMD), the significant alterations in circRNAs expression were evident (Dai et al., 2014; Zhao et al., 2017; Lee et al., 2019). In the case of cerebral ischemia-reperfusion injury (IRI), the upregulation of mmu-circRNA-015947 has been predicted and found in IRI patients, while the role of sponge mmu-circRNA-015947 is still unclear and require further studies (Liu et al., 2017; Lee et al., 2019).

The recent results have shown that circRNAs can regulate alternative splicing and modulate gene expression by sponging miRNAs. Therefore, the altered expression of circRNAs after SCI and other neurodegenerative diseases may be a key factor to modulate translation and protein production by competitively capturing miRNAs (Memczak et al., 2013; Li Z. et al., 2015; Salzman, 2016).

## circRNAs in SCI Neuroinflammation

Neuroinflammation is a complex process that varies with different types of injury and is promoted by proinflammatory factors released by resident glia, endothelial cells, and peripherally derived immune cells (Okada, 2016; Bloom et al., 2020). Following SCI, excessive neuroinflammation is considered as a key factor that contributes to neuronal damage. Recent studies have shown that circRNAs may participate in the modulation of neuroinflammation after SCI through chemokine/cytokine signaling pathways during the acute SCI (Chen X. et al., 2019; Xie et al., 2020). Li et al. (2020) reported that the down-regulated Circ0001723 related to miR-380-3p-HIF-1α induces pro-inflammatory effects 1 day after experimental SCI in rats, *via* NF-κB signaling pathway suppression, including the expression of NLPR3 inflammasome and caspase-1 proteins. *In vitro* SCI studies in rats have shown that the upregulation of Circ0000962 decreased inflammation, *via* down-regulation of miR-302b-3p and subsequent modulation of PI3K/Akt and NF-κB signaling pathways (He et al., 2020). The high expression of circRNA-2960 was able to promote the secondary injury by sponging the miR-124, exacerbating the inflammatory response and inducing apoptosis (Chen et al., 2021). Similar to SCI, the involvement of circRNAs in relation to neuroinflammation have also emerged by studying the acute ischemic stroke (AIS). Thus, it has been shown in both *in vitro* and *in vivo* cerebral ischemia models that the circ-HECTD1 knockdown regulates TRAF 3 (tumor necrosis factor receptor-associated factor 3) by miR-133b sponge in neuronal cells, improving cerebral infarction volume and neuronal apoptosis (Dai et al., 2021). In another report, it has been shown that circ-HECTD1 inhibits astrocyte activation both *in vivo* and *in vitro*, decreasing the infarct areas and neuronal deficits, due to circ-HECTD1 sponged miR-142 to upregulate TIPARP (tetrachlorodibenzo-p-dioxin inducible poly[ADP-ribose] polymerase), that inhibits astrocyte activation *via* autophagy (Han et al., 2018b). Besides, upregulation of circDLGP4 has been shown to attenuate infarct areas and blood-brain barrier damage in the transient middle cerebral stroke (_t_MCAO) model (Bai et al., 2018). In summary, these studies revealed that dysregulated circRNAs are closely correlated to immune reactions, which makes them possible therapeutic targets to modulate neuroinflamation and neuronal death after SCI and other brain diseases.

## circRNAs as Biomarkers

It is obvious that circRNA can regulate CNS function in health and disease and that they are potential biomarkers for the diagnosis and prognosis of different CNS diseases and disorders, alone or in combination with other biomarkers and imaging tools (Lu and Xu, 2016; Han et al., 2018a; Zhang et al., 2018; Xie et al., 2020). In addition to being considered as possible therapeutic targets to promote recovery after injury, circRNAs from blood or cerebrospinal fluid are suggested as potential non-invasive tools to indicate cellular damage after CNS injury or disease and are thus good candidates as biomarkers, due to their high stability, abundance and specificity in CNS (Li Y. et al., 2015; Lu and Xu, 2016; Han et al., 2018b; Xu et al., 2021). However, more studies are required to explore the way in which the exo-circRNAs participate at different stages of SCI to enable their use as biomarkers in SCI diagnosis and prognosis. Since neuroinflammation is a common pathophysiology process occurring in many different CNS disorders and diseases, including SCI, ischemic stroke, and neurodegenerative diseases (Lu and Xu, 2016), further studies on the distribution and transcriptional profiles of the circRNAs and comprehensive circRNAs expression profile analysis are needed to find shared or different circRNA changes. Only then, particular circRNAs could be identified as potential clinical biomarkers for specific CNS disorders and conditions, changing specific expression only in certain circumstances.

## Pharmacological Interventions and circRNAs

It has been shown that pharmacological interventions can seriously modulate circRNAs expression in brain and spinal cord, and subsequently lead to complex compensatory changes, including drug tolerance. For example, recent studies demonstrated that prolonged use of opioids can alter circRNAs expression in the spinal cord of morphine tolerated rats and that changed circRNAs were related to glutamatergic transmission, MAPK signaling pathway, and axon guidance (Weng et al., 2019). Furthermore, it was shown that several circRNAs binding sites at opioid receptor gene (Oprm1) are involved in modulation of circRNAs expression after chronic morphine treatment in the brain and spinal cord of adult male CD-1 mice (Irie et al., 2019). Other opioid receptor genes including δ, κ, as well as nociceptin receptor genes can also be involved in circRNAs metabolism after chronic morphine treatment (Irie et al., 2019). Recently, an interesting study of regulatory networks using graphene quantum dots, a novel bio-imaging and bio-sensing delivery system for novel drugs, could be an approach with the potential to be used also to study the role of circRNAs in signaling pathways related to toxicity and inflammation (Wu et al., 2021). These data open the need for further investigations of the changes in circRNAs expression induced by drugs in the CNS, creating the possibility of interfering with the pharmacodynamics of neuromodulatory drugs which could also be used to treat SCI.

## Conclusions

**Figure 1:**
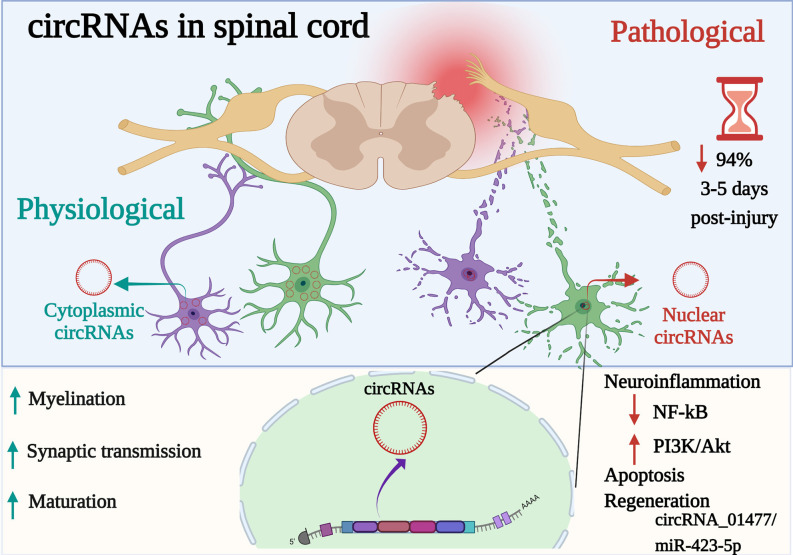
Participation of circRNAs as key active regulators in diverse physiopathological mechanisms in the spinal cord. circRNAs are a special type of endogenous ncRNAs formed by back-splicing events *via* protein-coding exons. Differentially expressed circRNAs (cytoplasmic or nuclear) are implicated in biological, cellular, molecular processes and gene expression modulation at post-transcriptional level in the spinal cord. Whereas, dysregulated circRNAs (up or downregulated) have been associated with acute stages following spinal cord injury (SCI), acting as a sponge of miRNAs, suppressing or activating signaling pathways related (e.g., NF-κB, PI3K-AkT) to signal transduction, neuroinflammation, and apoptosis but to promote regeneration. Indeed, even if 94% of cirRNAs decreased after 3–5 days post-spinal injury, circRNA_01477/miR-423-5p is a key circRNA to control regeneration. Created with Biorender.com.

SCI causes life-long disability which often results in high rehabilitation costs and reduced patient’s quality of life. No current effective therapies for SCI are available, in part because of limited understanding of molecular events underlying SCI. Also, the biomarkers to follow injury severity are lacking. circRNAs are ncRNAs that interact with different target genes and proteins that are dysregulated after SCI with the possible consequences on different cellular and molecular processes, including neuroinflammation, leading to neuronal death or cell survival (Figure 1). The contribution of circRNAs and their functions in the critical stages of SCI have barely been identified using *in vitro* and or *in vivo* animal models. The high abundance in body fluids make circRNAs potential non-invasive clinical biomarkers to improve diagnosis and prognosis after SCI, as well as they represent possible targets for the development of new SCI therapeutic strategies.

## Author Contributions

CS and GM contributed to the conception of principal ideas, wrote the first draft of the manuscript and performed the graphic design. MM wrote some sections of the manuscript, and thoroughly proofread the last version. All authors contributed to the article and approved the submitted version.

## Conflict of Interest

The authors declare that the research was conducted in the absence of any commercial or financial relationships that could be construed as a potential conflict of interest.

## Publisher’s Note

All claims expressed in this article are solely those of the authors and do not necessarily represent those of their affiliated organizations, or those of the publisher, the editors and the reviewers. Any product that may be evaluated in this article, or claim that may be made by its manufacturer, is not guaranteed or endorsed by the publisher.
